# Challenges in the Design and Fabrication of a Lab-on-a-Chip Photoacoustic Gas Sensor

**DOI:** 10.3390/s140100957

**Published:** 2014-01-08

**Authors:** Alain Glière, Justin Rouxel, Mickael Brun, Bertrand Parvitte, Virginie Zéninari, Sergio Nicoletti

**Affiliations:** 1 CEA, LETI, MINATEC Campus, 17 rue des Martyrs, Grenoble F-38054 Cedex 9, France; E-Mails: justin.rouxel@cea.fr (J.R.); mickael.brun@cea.fr (M.B.); sergio.nicoletti@cea.fr (S.N.); 2 Groupe de Spectrométrie Moléculaire et Atmosphérique, Unité Mixte de Recherche 7331, Centre National de la Recherche Scientifique, Faculty of Science, Reims University, Moulin de la Housse, Reims F-51687 Cedex 2, France; E-Mails: bertrand.parvitte@univ-reims.fr (B.P.); virginie.zeninari@univ-reims.fr (V.Z.)

**Keywords:** lab-on-a-chip, miniaturization, model, photoacoustic spectroscopy

## Abstract

The favorable downscaling behavior of photoacoustic spectroscopy has provoked in recent years a growing interest in the miniaturization of photoacoustic sensors. The individual components of the sensor, namely widely tunable quantum cascade lasers, low loss mid infrared (mid-IR) waveguides, and efficient microelectromechanical systems (MEMS) microphones are becoming available in complementary metal–oxide–semiconductor (CMOS) compatible technologies. This paves the way for the joint processes of miniaturization and full integration. Recently, a prototype microsensor has been designed by the means of a specifically designed coupled optical-acoustic model. This paper discusses the new, or more intense, challenges faced if downscaling is continued. The first limitation in miniaturization is physical: the light source modulation, which matches the increasing cell acoustic resonance frequency, must be kept much slower than the collisional relaxation process. Secondly, from the acoustic modeling point of view, one faces the limit of validity of the continuum hypothesis. Namely, at some point, velocity slip and temperature jump boundary conditions must be used, instead of the continuous boundary conditions, which are valid at the macro-scale. Finally, on the technological side, solutions exist to realize a complete lab-on-a-chip, even if it remains a demanding integration problem.

## 1. Introduction

Photoacoustic (PA) spectroscopy is a well-established technique and numerous gas sensors designs, relying on a single cavity, based on Helmholtz resonance, or enhanced by quartz tuning forks have been imagined and implemented [1]. However, the mass deployment of PA gas sensors is hampered by the systems' overall size and weight, and their high cost of ownership and maintenance. The availability of a lab-on-a-chip (LOC)-based solution for multigas analysis could open the way to the low-cost market and to applications such as indoor air monitoring, air pollution control, or greenhouse gases measurement.

In PA spectroscopy, the modulation of a light source creates a time-periodic variation of temperature due to non-radiative relaxation of excited molecules, and thus an acoustic wave in the chamber. The use of laser sources in the mid infrared (mid-IR) wavelength range, from 3 to 12 μm, maximizes the light absorption by a number of molecules of interest for a wide variety of applications. The combination of PA detection with mid-IR pumping is particularly adapted to the detection of chemicals in trace amounts.

The acoustic signal is inversely proportional to the volume of the resonant cell [2]. This favorable downscaling behavior has provoked in recent years a growing interest in the size reduction of PA cells. One can particularly cite the pioneering work of Firebaugh [3], where a ∼4 mm^3^ trapezoidal chamber, etched in silicon, was capped with a silicon membrane microphone. The sensor was able to detect 10 ppm of propane in nitrogen. In the same vein, Pellegrino and Holthoff [4], addressing the detection of chemical agents, designed and characterized a differential cell of ∼15 mm^3^ internal volume, reaching a detection limit of ∼20 ppb for dimethyl methylphosphonate. Other notable progress in the size reduction direction has also been performed by Gorelik *et al.* [5], with inclined geometry cells (∼500 mm^3^ internal volume), who reached, for instance, a detection limit of ∼10 ppm for ammonia. On their side, Karioja *et al.* [6] implemented a low-temperature co-fired ceramics technology to build a ∼8 mm^3^ differential PA cell. However, it seems that no gas detection measurements results obtained with this tiny sensor have been published. Very recently, Rueck *et al.* [7] have initiated the process of using microelectromechanical systems (MEMS) technologies to combine a ∼12 mm^3^ cavity etched into a glass wafer and a piezoelectric cantilever microphone.

All the individual components of the PA sensor, such as widely tunable quantum cascade laser (QCL) sources [8,9], low loss mid-IR waveguides [10,11] and efficient MEMS microphones [12,13] are becoming available in complementary metal–oxide–semiconductor (CMOS) compatible technologies. Thus, in order to produce small sensors, requiring no optical setting, the joint processes of miniaturization and full integration in MEMS technologies of a PA cell working in the mid-IR range have been initiated [14]. It is worth noting that the integration of the laser and mid-IR photonic circuitry remains out of the scope of the previously cited approaches [3–7].

Among the various PA sensors principles available, the differential Helmholtz resonator (DHR) [15] is investigated in this work. The DHR consists of two identical chambers connected by two capillaries. Although only one chamber of the sensor is illuminated by a laser beam, acoustic waves are established in both chambers. The signals from microphones measuring the pressure in each chamber, opposite in phase at the resonance frequency, are subtracted by a differential amplifier. This results in increasing the useful signal while the in-phase external acoustic noise is partially cancelled out. The DHR principle has been chosen because many of its features turn into advantages during the miniaturization process. Firstly, the sensor is relatively insensitive to the shape of the energy deposition localization because the overlap integral of the fundamental mode is almost constant in the illuminated chamber. As confirmed by simulation results [14], this mitigates the effect of the strong divergence of the laser beam at its entrance into the chamber. Second, as the pressure is constant in each chamber, it is easy to place several microphones by chamber to improve the signal to noise ratio of the sensor. Third, as the value of the quality factor of the cavity is small, the uncertainty on the microphones resonance frequency, due to fabrication variation, is unimportant. Finally, as the cell is symmetrical, the gas input and output can be plugged into the middle of the capillaries, where pressure nodes are located. The effect of the associated dead volumes is thus mostly cancelled.

**Figure 1.**
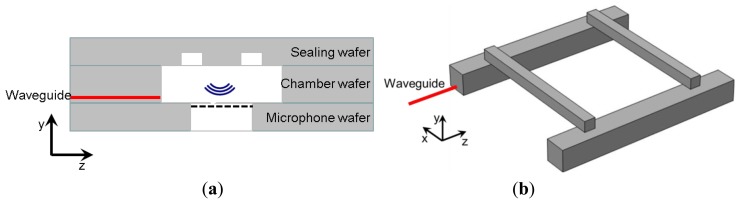
Schematic view of (**a**) the μ-PA DHR cell constituted by a stack of three wafers and (**b**) CAD model of the cavity. The hollow part is white in the left figure and grey in the right figure.

In a previous article [14], a coupled optics-acoustics model dedicated to the simulation of miniaturized and integrated PA gas detectors has been presented. Using this model and taking into account the design rules of MEMS technologies, a miniaturized DHR cell has been devised (Figure 1). This μ-PA sensor is composed of three different wafers, assembled by eutectic bonding. The MEMS microphones are built independently in the first wafer. The mid-IR waveguides are created by epitaxy, delimited by etching, and then buried under a thin layer of silicon [11] in the second wafer. Afterwards, the same second wafer is thinned to the desired chamber thickness (300 μm) and, finally, the two chambers are etched across it. The two capillaries are etched in the third wafer, which also constitutes the ceiling of the chambers. The total cell volume is less than 0.6 mm^3^.

A significant downsizing step has thus been achieved. However, the question of the dimensions at which the full potential of miniaturization will be obtained still remains open. In this paper the consequences of further miniaturization on the physics, models and microfabrication technology are discussed from a theoretical point of view. In the first part of the paper, two aspects of the consequences of downsizing on the models are investigated. Namely: (i) the dynamics of gas molecule excitation by modulated IR light and relaxation is studied; and (ii) the applicability of the continuum model hypothesis to ever shrinking gas sensors is re-evaluated. In the last part of the paper, an overview of the MEMS fabrication challenges and the resulting constraints on the microdevice design is presented. Even though the focus is placed here on the μ-PA cell currently under fabrication, our expectation is to provide as general insights as possible.

## 2. Challenges in Modeling and Simulation

It is not economically viable to build and test a large number of different DHR cells since the technological steps required for their fabrication are expensive and time consuming. Resorting to modeling and numerical simulation to optimize the performance of the sensor is a way to partially overcome these limitations. However, one should note that a global design methodology, iteratively adapting the simulation tools with the fabrication process flow, is necessary. In fact, at the micro-scale, even more than at the macro-scale, the choices of fabrication methods and the constraints imposed on the device dimensions drive new model developments, while modeling results help exploring novel routes for the technological implementation.

Due to the very diverse domains of physics involved, the model should address: (i) the mid-IR electromagnetic mode propagation in the waveguide; (ii) the light illumination of the chamber; (iii) the interaction of light with the molecules of interest and the relaxation process; (iv) the creation and propagation of acoustic waves in the cell; and finally (v) the acoustic wave sensing by the microphone. Many of these physics are intimately coupled but, for the problem to remain tractable, advantage should be taken of all the uncoupling possibilities, such as that occurring because of the linear dependence of the signal on the deposited energy.

### 2.1. Downscaling of the Optical Model

The optical model generally used for PA sensors assimilates the laser illumination geometry to a straight beam for which the flux follows a Gaussian distribution within a cross section [16,17]. This basic laser beam model is well adapted at the macro-scale but collapses when the PA cell is miniaturized and integrated in planar silicon substrates. Several issues arise. First, in a LOC configuration, the mid-IR radiation should be injected in the chamber by the means of a waveguide, whose section's dimensions are of the order of the light wavelength. At the exit section of the waveguide, the beam is diffracted and diverges strongly. Second, silicon is transparent to mid-IR radiation. Thus, the natural confinement of energy obtained by reflection on the usually metallic walls of macro-size devices is not present. Moreover, in the DHR configuration, a portion of the light emitted by the laser source is refracted towards the non-illuminated chamber and interacts there with the gas. This crosstalk between chambers can adversely affect the detector performance.

A new optical model, briefly recalled here, has been devised to cope with the above mentioned problems [14]. The model is made up of two parts, respectively accounting for the propagation of the electromagnetic field in the waveguide and in the chamber.

In the μ-PA device, a QCL operating in transverse magnetic polarization is coupled to a SiGe/Si waveguide, designed to be monomodal at the mid-IR wavelength of interest [10]. The fundamental mode propagating in the waveguide can be calculated by the finite elements method (FEM). The resulting mode is then injected in the second part of the optical model, which handles the light propagation in the chamber.

The length scale ratio between the size of the illuminated chamber (several millimeters long) and the mid-IR wavelength (∼3–12 μm) is too large for practical solution of the Maxwell's equations by exact full-wave methods, such as the finite difference time domain method or the finite element method. Nevertheless, in this regime, the propagation of light can be modeled by geometric optic tools, which are less computationally demanding, while providing almost as accurate results. The raytracing method relies on the plane wave decomposition of the electromagnetic source (*i.e.*, the exit section of the waveguide). It is an approximate solution of the Maxwell's equations, which only takes into account reflection and refraction at locally plane interfaces, and fails if light diffracting sub-wavelength features are present in the system. The method of choice is the combination of raytracing with the Monte Carlo method [18]: a large number of rays are followed individually from their random generation at the light source to their exit of the computational domain. Here, the plane wave decomposition of the source is obtained by Fourier transform of the electric field map of the guided mode. The Fourier transform is performed analytically if the electric field can be fitted to a two-dimensional Gaussian function, or numerically otherwise. The energy carried by each ray is partially absorbed along its path due to interactions with gas molecules. Assuming that the whole energy of the absorbed photons is released locally as heat [2], the three-dimensional map of the energy deposition is computed with the Beer-Lambert law and recorded for use by the acoustic model. The non-sequential raytracing module of the commercial software Zemax (Radiant Zemax, Redmond, WA, USA) is used.

**Figure 2.**
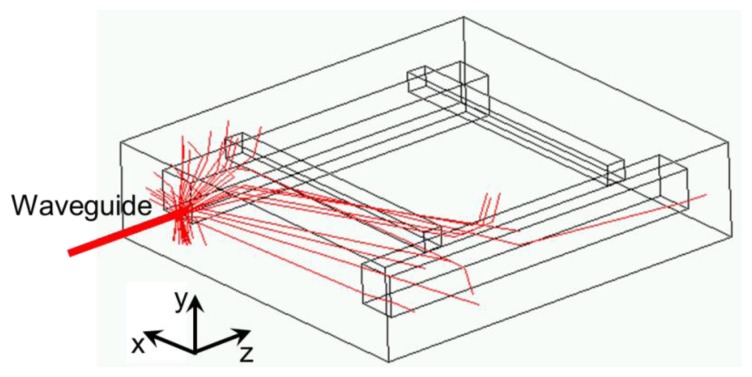
Raytracing in the μ-PA DHR cell. Crosstalk appears when light emitted by the laser source is refracted towards the non-illuminated chamber, where it can interact with the gas.

An illustration of raytracing, involving only a few rays for clarity, is presented in Figure 2. The energy absorbed in the illuminated chamber is mostly located close to the waveguide exit and a part of the energy, of the order of several percent, is deposited in the non-illuminated chamber [14].

### 2.2. Molecular Relaxation

In photoacoustic spectroscopy of gases, intensity or frequency modulated light from the laser enters the measurement cell filled with the gaseous sample. A portion of the incident radiation is absorbed by the gas resulting in a pressure disturbance. When a gas molecule absorbs a photon, it goes from its ground state to an excited state, the energy difference between the states being the energy of the absorbed photon. In a subsequent step, the molecule loses this excess energy and returns to the ground state in one among several ways: radiative deexcitation, intersystem energy transfer, and energy transfer by collision with other molecules [19]. The sound wave detected by the microphone results from the third process, which consists in heating and dominates in the mid-IR range [2]. If the incident photon radiation is modulated at a rate that is slow compared to the rate of this process then the optical modulation results in a coherent modulation in the temperature of the gaseous sample. On the opposite, if the modulation frequency is too high, not all the absorbed energy appears as periodic heat, and the phase and amplitude of the photoacoustic signal can be noticeably different.

In the models used at the macro-scale, it is assumed that: (i) the absorbing molecular transition is not saturated; and (ii) the relaxation time is much smaller than the period of the source modulation [16]. As the cell size reduction goes along with changes in laser power density and with an increase of the resonance frequency, and thus the modulation frequency, the validity of these hypotheses should be checked again.

The rate equation approach is adequate to study the joint effects of absorption and excited molecules relaxation, and therefore delineate the different operating regimes of the heat source. The analysis made by Kreuzer [20,21] for a two-level system is followed here. It is assumed that: (i) the light density is uniform and covers the whole chamber; (ii) the only path for relaxation of the upper state population are radiative and collisional; and that (iii) collisional excitation does not occur. The following global rate equation for the density *n* of absorbing molecules in the excited state is obtained:

(1) $$\frac{dn}{dt}=-n(\frac{I}{hv}\sigma +\tau^{-1})+(N-n)\frac{I}{\text{h}v}\sigma$$

In this equation, *I* is the time dependent beam intensity, *hν* the photon energy, σ the absorption cross-section of the molecules, τ the relaxation time, and *N* the total density of absorbing molecules. In the infrared region, the radiative relaxation time is much larger than the collisional one [2] and, hereafter, τ only accounts for collisional relaxation. The heat source density is given by:

(2) H(t)=hvn/τ

The discussion of Kreuzer [21] is oriented towards the limiting cases but it is possible to obtain wider insight by taking advantage of the numerical solution of the differential equation. To begin with, the non-dimensional version of Equation (1) is derived:

(3) $$\frac{d\tilde{n}}{d\tilde{t}}=-N_{1}\tilde{n}+N_{2}\frac{1}{2}(1-\cos(2\pi \tilde{t}))(1-2\tilde{n})$$

The characteristic scales used for the density of excited molecule *ñ* is *N* and that used for time t̃ is the inverse 1/*f* of the modulation frequency. Two non-dimensional numbers, *N*_1_=1/(*f*τ) and *N*_2_=σ*I*/(*f*h*v*), respectively represent the relationship between the modulation period and the relaxation time, and between the modulation and the absorbed photon flux. For simplicity, a harmonic intensity modulation of the laser is assumed. Let us note that the heat source is proportional to *ñ*.

**Figure 3.**
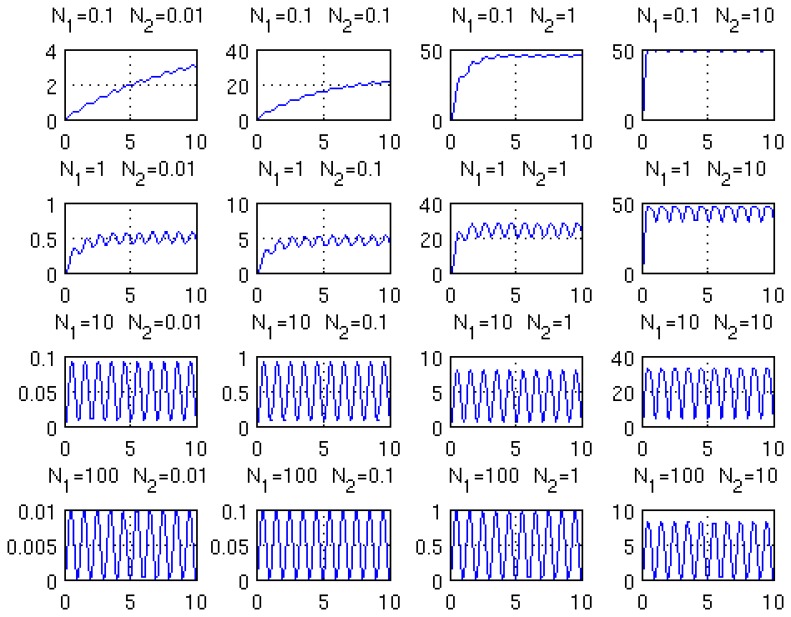
Plot of the non-dimensional excited molecules density *ñ*, expressed in per cents, versus non-dimensional time for several values of *N*_1_ (0.1, 1, 10 and 100, from top to bottom) and *N*_2_ (0.01, 0.1, 1 and 10, from left to right).

The results of the solution of Equation (3), for several combinations of the non-dimensional numbers *N*_1_ and *N*_2_, are presented in Figure 3. When the value of *N*_1_ decreases, the molecule relaxation cannot cope with the too fast excitation and a part of the modulation amplitude is lost. When the value of *N*_2_ increases, the amount of available non-excited molecules decreases and at some point saturation occurs.

**Figure 4.**
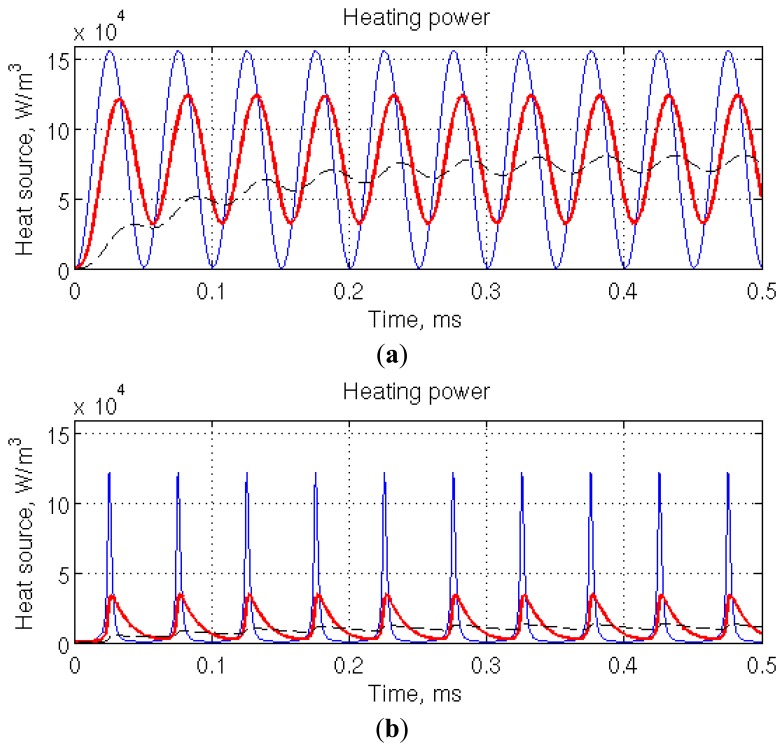
Time evolution of the heat source density in typical μ-PA conditions. The relaxation rate time constant is 1 μs (blue thin line), 11 μs (red thick line), and 100 μs (black dashed line). (**a**) Amplitude and (**b**) wavelength modulation.

The typical working point of the μ-PA cell (*N*_1_∼5 and *N*_2_∼0.02) lies in the left part of Figure 3, where saturation is not effective. This is emphasized in Figure 4a, in which computation conditions are representative of the foreseen μ-PA cell (20 kHz amplitude modulation, laser power 1 mW, illuminated chamber cross-section 300 μm × 300 μm) and of the absorption characteristics of carbon dioxide in standard atmosphere at 4.2 μm (peak absorption cross section: 1.42 × 10^−17^ cm^2^/molecule from HITRAN database [22], concentration: 397 ppm). The rate equation is solved for three different values of the relaxation time constant spanning three orders of magnitude. At the intermediate value, 11 μs, corresponding to the relaxation time of carbon dioxide in nitrogen [23], around 40% of the heat source modulation is lost. For 100 μs, more than 90% of the modulation is lost. These results are consistent with the (1+ ω^2^τ^2^)^−1/2^ dependency obtained by analytical analysis [24]. The modulation loss is accompanied by a phase shift, also visible on the figure. The model has been further adapted to non-harmonic wavelength modulation (Figure 4b), by assuming a sinusoidal scan of the absorption peak of interest, modeled by a Lorentzian function, and no simultaneous amplitude modulation. The intensity, peak frequency and width of the Lorentzian function are excerpted from the HITRAN database. The modulation loss is more pronounced as, for 11 μs relaxation time, only about 25% of the modulation is kept.

The previous computations have been performed assuming a uniform illumination of the chamber. However, in the μ-PA case, due to beam divergence, the latter is highly variable throughout the illuminated chamber. Thus, it would be useful to get the more precise picture obtained with a local rate equation formulation. The local conservation equation can be derived from Equation (1) by replacing *n*(*t*) and *I*(*t*) by their space dependent counterparts *n*(**r**,*t*) and *I*(**r**,*t*). Let us first note that the two contributions corresponding to the diffusion of the excited molecules and their convection by the acoustic velocity field can be neglected. Indeed, on the one hand, assuming that the excited and non-excited gas molecules behave similarly in the host gas, no concentration gradient, and thus no diffusion flux, is present. On the other hand, based on FEM computation in the μ-PA configuration, the acoustic velocity can be estimated around a few millimeters per second. In the case of carbon dioxide in air, during the excited state lifetime, the distance traveled by convection is in the tens of nanometers range. Convection can thus be safely neglected as the energy deposition zone is three orders of magnitude larger.

If the generally adopted assumption that the absorption of the mid-IR flux by the gas molecules is low enough, is valid, the local flux density *I*(**r**,*t*) can be provided by the raytracing software. Then, the space dependent differential equation can be solved, and the local heat source density *H*(**r**,*t*) = *n*(**r**, *t*) *hv*/τ can finally be injected in the acoustic solver. An algorithm coupling in sequence the raytracing model, the rate equation, and the acoustic model can thus be derived. In the other case, where relative absorption is too important, the raytracing and absorption models must be strongly coupled. In any case, the interest of developing this sequentially coupled model should be assessed.

It should also be noted that the relaxation time vary on several orders of magnitude (see for instance Table 1 for the relaxation times of several simple molecules on nitrogen) and that the composition of the gaseous sample can introduce additional relaxation ways, either due to the studied molecule itself or to the presence of other gases in the sample [19]. For instance, relaxation of ozone is not direct from the excited state to the ground state [25], or water vapor concentration can have a favorable influence on carbon dioxide measurement [23].

The real picture is thus more complex than the simple case of the intensity modulated two-level system with uniform illumination computed above but the broad lines of the reasoning are still valid. It appears that (i) the heat source modulation loss is significant for a cell resonating at 20 kHz; and that (ii) the modulation will further decrease with miniaturization, as resonance frequency will increase. In fact, set apart quartz enhanced photoacoustic spectroscopy [29], based on wristwatch quartz tuning fork functioning around 30 kHz, and the small size sensor developed by Holthoff *et al.* [4,30], the PA sensors modulation frequency is generally less than 4 kHz [1]. This suggests a trade-off in miniaturization as the expected signal improvement is, at least partly, cancelled out by the heat source modulation loss.

### 2.3. Downscaling of the Acoustic Model

The pressure acoustic model is commonly used to study PA device behavior. Assuming adiabatic propagation in an ideal lossless gas, it consists of a single inhomogeneous Helmholtz equation for the unknown pressure [31].

However, various volume and surface dissipation processes are at work in the gas, in the bulk of the propagation medium and close to the cell walls, respectively [2,31]. The latter are of utmost importance in miniaturized PA devices. They occur by viscous dissipation and heat conduction in thin boundary layers located near the cell walls. In the interior of the cell, the gas acoustic velocity is proportional to the pressure gradient whereas at the wall, the no-slip boundary condition imposes a null tangential component. Thus, viscous dissipation occurs in a transition region, the viscous boundary layer. Similarly, the thermal boundary layer is the transition region where the adiabatic expansion and contraction of gas occurring in the interior of the cell, turns to isothermal, due to the high thermal conductivity of the cell walls relative to that of the gas.

**Figure 5.**
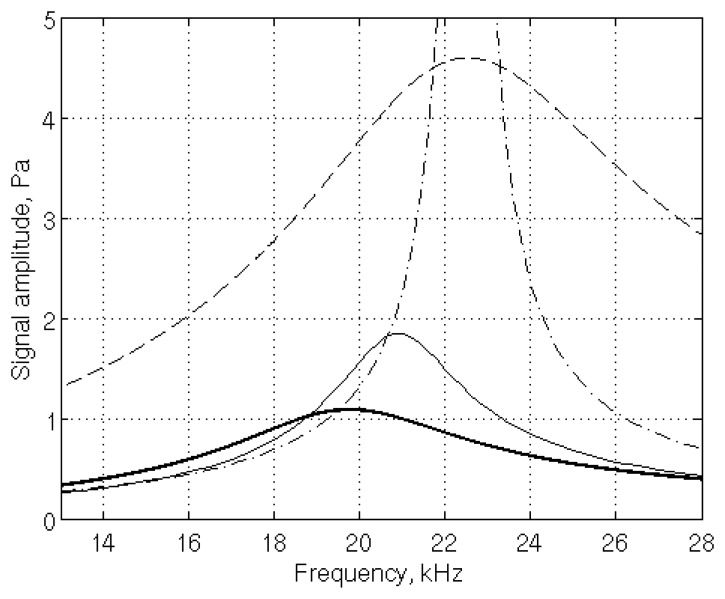
Frequency response of the μ-PA cell obtained with four different models: pressure acoustic (dash-dotted line), pressure acoustic with correction for dissipation effects by quality factors (dashed line) or boundary conditions (thin line), and viscothermal model (thick line).

Approximate models can be adapted from the pressure acoustic model to take into account the dissipation effects, for instance with eigenmode expansion and introduction of quality factors [16], or with especially designed boundary conditions [17]. These models are computationally efficient and accurate at the macro-scale [32], but fail to correctly represent miniaturized cells, where boundary layers occupy a non-negligible part of the capillaries (Figure 5). In a miniaturized DHR device, such as the one studied here, with a working frequency around 20 kHz, in air at standard temperature and pressure, both boundary layer thicknesses are in the 15 μm range, which is of the same order of magnitude as the capillary side length.

A viscothermal model [33,34], directly derived from the first principles governing equations, namely, the mass, momentum, and energy conservation laws supplemented with a thermodynamic equation of state, is an efficient alternative. Coupled with the optical model described in Section 2.1, it has been harnessed to design the first generation of the μ-PA sensor prototype [14].

The necessity to use the sophisticated, but computationally demanding, viscothermal model is illustrated in Figure 5, where its results are compared with those obtained with the pressure acoustic model without correction for dissipation effects and with correction by two different methods [16,17]. On the one hand, as expected, the pressure acoustic model (dash-dotted line) is of limited use: as no dissipation mechanism is accounted for, the peak signal value is unbounded. On the other hand, both modified models overestimate the expected resonance frequency and signal. The model involving adapted boundary conditions provides more accurate resonance frequency and signal value. It could be useful to obtain fast, even if approximate, results.

### 2.4. Validity of the Continuum Model

It has been assumed up to this point, that the gas contained in the μ-PA chamber, although composed of a myriad of rapidly moving and colliding molecules, is a continuous medium, represented by locally averaged macroscopic quantities, such as density, pressure, and temperature. These macroscopic quantities are defined as local averages on fluid elements that, ideally, are large enough to contain a considerable number of molecules, typically 10^6^, but small enough to permit the use of differential calculus. In these conditions, statistical fluctuations of the macroscopic quantities are not significant (<0.1%) and molecular chaos conditions are achieved due to frequent collisions. The macroscopic quantities vary continuously in space and time, and are governed by the mass, energy, and momentum conservation laws. These equations, referred to as the Navier-Stokes equations in the following text, can be handled by numerical methods, such as the FEM or the finite volume method. However, as downscaling is carried on, it is natural to investigate the domain of validity of the continuum framework. The brief discussion that follows is inspired from the microfluidics [35] and MEMS [36] classical literature. It is limited here to gas flows and especially focused on the μ-PA device working conditions.

In statistical mechanics, the starting point is the Liouville equation, which expresses the conservation of the particle distribution function in the phase space, consisting in all the possible values of the position, momentum and internal states of all the molecules. Due to the large number of particles (*N*) and dimensions of the phase space (6*N* for monoatomic gases), the Liouville equation cannot be used to simulate any practical engineering problems. However, assuming dilute gas and molecular chaos, the Boltzmann transport equation can be derived from the Liouville equation. Dilute gases are those where the mean molecular spacing *δ* is much larger than the gas molecule diameter *d*. In these conditions, binary intermolecular collisions are much more likely than simultaneous collision of several particles.

Additionally, if the fluid is close enough to thermodynamic equilibrium, the Navier-Stokes equations can be used. The frequency of collisions in the gas bulk, a measure of the proximity to thermodynamic equilibrium, is governed by the Knudsen number, *Kn*, defined as the ratio between the molecules' mean free path λ and a characteristic length of the flow *L*. The chosen characteristic length can be a geometric dimension of the device under consideration or, as in our case, the thickness of a boundary layer across which macroscopic quantities experience significant gradients.

Special attention must be taken when approaching the fluid-solid interface. If the frequency of collisions at the chamber wall is high enough, the thermodynamic equilibrium is established between the gas and solid particles. Continuity of particles velocity and temperature at the fluid-solid interface respectively lead to the no velocity slip and no temperature jump boundary conditions. However, if the collision frequency is not high enough, the thermodynamical equilibrium is not established. Tangential velocity slip and temperature jump, ruled by tangential momentum and thermal accommodation coefficients (σ*_v_* and σ*_T_*), must be taken into account Equations (4) and (5). The accommodation coefficients depend on the interface nature and surface condition:

(4) $$u_{\text{gas}}=\frac{2-\sigma_{v}}{\sigma_{v}}\lambda \frac{\partial u}{\partial n}$$

(5) $$T_{\text{gas}}-T_{\text{wall}}=\frac{2-\sigma_{T}}{\sigma_{T}}\frac{2\gamma }{\gamma +1}\lambda \frac{\kappa }{\mu C_{p}}\frac{\partial T}{\partial n}$$

For simplicity, in Equation (4), the wall has been assumed motionless and the thermal creep term, irrelevant as long as the wall is considered isothermal, has been omitted.

**Figure 6.**
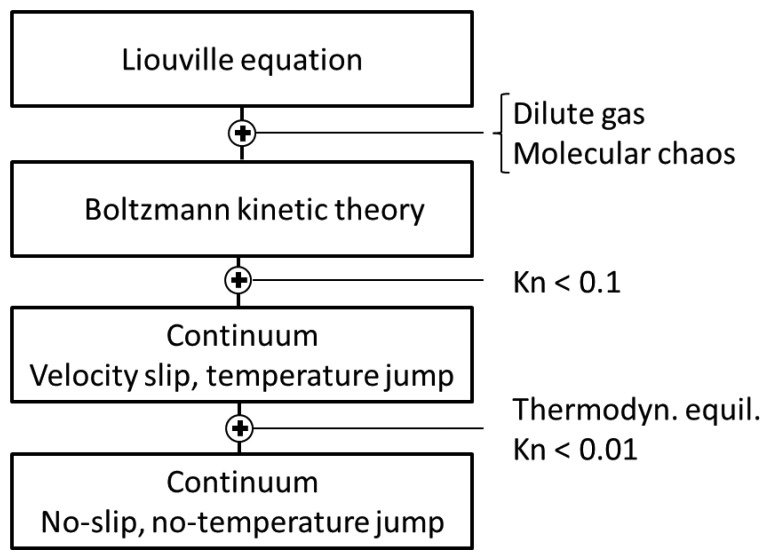
Summary of available molecular and continuum flow models.

A summary of the molecular and continuum flow models and the major hypotheses allowing passing from a model to the next one is presented on Figure 6. While molecular approaches are harnessed for computing gas flows in methods such as direct simulation Monte Carlo, only the continuum hypotheses based equations are usable to deal with realistic engineering problems and geometries.

**Figure 7.**
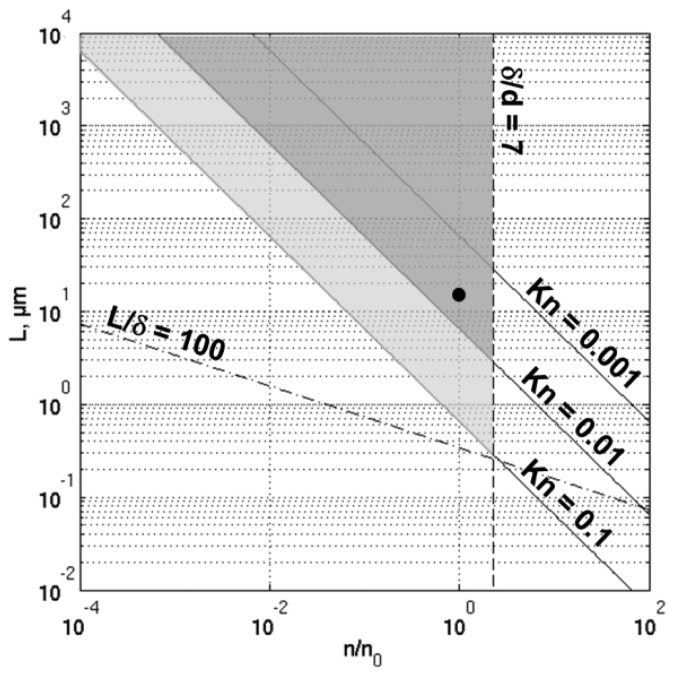
Domain of validity of the continuum approximation without (dark grey triangle) or with (light grey area) velocity slip and temperature jump boundary conditions, and typical working point of the μ-PA cell (●). Figure inspired from Karniadakis and Beskok [35] and Gad el Hak [36].

The domain of validity of the continuum approximation, with continuous boundary conditions, is represented, in Figure 7, by the grey triangle delimited by the straight lines corresponding, respectively, to the continuum flow hypothesis (*Kn* ≲ 0.01) and the dilute gas approximation (δ/*d* ≳ 7). The plot space is constituted by the non-dimensional number of molecules per unit volume (*n*/*n*_0_) and the characteristic length scale of the gas flow L. The number of molecules per unit volume at standard temperature and pressure is denoted *n*_0_. When improved by velocity slip and temperature jump boundary conditions, the Navier-Stokes equations can be used in the whole light grey area, delimited by the *Kn* = 0.1 line. Let us note that: (i) these limits result from empirical considerations; (ii) they are configuration dependent; and (iii) some authors claim that velocity slip and temperature jump boundary conditions should be used as soon as *Kn* ≳ 0.001. The *Kn* = 0.1 line delimits the transitional flow regime, where the linear constitutive laws for stress tensor and heat flux, and thus the whole Navier-Stokes equations framework fails. Beyond this limit, either higher correction of the constitutive laws or the Boltzmann equation must be dealt with.

A typical working point of the μ-PA cell is placed in the diagram (Figure 7). The chosen length scale is the thickness of the viscous boundary layer (*d*_μ_ ≈ 15 μm) and the molecular diameter of air is set to 0.37 nm [35]. The characteristics of the working point are summarized in Table 2. From the diagram, it can be noticed that:

- The working point is far above the insignificant statistical fluctuations limit (*L*/δ ≳ 100). This is true even if we consider that, for this particular topic, the length scale should rather be the thickness of the finite element layer close to the cell's walls (it is advisable to place at least two or three elements in the viscous boundary layer thickness).
- The working point is located close to the limit where the use of velocity slip and temperature jump boundary conditions becomes compulsory.
- The reduction of the device size, going along with the increase of the resonance frequency, and thus the decrease of the boundary layer thickness would bring the limit even closer. The same effect would be reached by the reduction of the working pressure.

## 3. Technological Challenges

Modeling seems to validate the approach of using PA cells of sub-millimeter dimensions but shrinking the size of the cell at this level remains a technological challenge. Although precision machining has been successfully used to realize absorption cells several millimeters long [4], this option is not suitable to push the miniaturization further. Moreover, miniaturization will be effective not only by reducing the size of the cell itself, but also that of the optical source and of the acoustic detection means. Microelectronics and MEMS manufacturing facilities mix together all the capabilities to realize such an object, bringing furthermore very good reproducibility, low cost, and large scale manufacturing. Co-integration, in a single silicon chip, of a mid-IR optical source, a waveguide set for beam handling, and a photoacoustic cell with MEMS microphones can thus be envisioned to target a fully integrated sensor. Nevertheless, many technological obstacles must be overcome to reach this goal.

Regarding the realization of the PA cavity itself, silicon manufacturing technologies come with design rules which impact directly the shape and size of the acoustic cavity. For example, lithography and deep reactive ion etching processes lead to vertical walls geometry, with constrained aspect ratio, restricting the shape of the cavities to parallelepipedic volumes. It must also be noted that it is not straightforward to realize two connected volumes of different depths in a single wafer without creating non-desired over etched zones. This can be detrimental to realize a DHR cell, for which the capillaries need to be much smaller in depth than the chambers. Thus, integrating the chambers and the capillaries in a single silicon wafer is challenging. To a certain extent however, it is possible to stack two or more wafers together by using eutectic sealing or wafer bonding, using a metallic or polymer layer. This technique allows processing separately the integrated MEMS microphones, whose complex fabrication can be developed and stabilized independently. One possible resulting configuration is depicted in Figure 1 with a MEMS microphone in a first wafer, PA chambers and mid-IR waveguides in a second one, and capillaries in a third wafer. The three wafers are bonded together to complete the LOC. In this design, as the cavity is etched across a wafer, its depth is limited on the one hand by the thickness of the standard silicon wafer (725 μm for 200 mm wafers) and on the other hand by the difficulty in manipulating too thinned wafers (≲300 μm).

Several MEMS microphones types are nowadays available for sensing the acoustic pressure within the cavity. Generally they rely on the realization of a self-standing membrane, able to oscillate under the effect of the acoustic pressure wave. The amplitude of vibration is converted into an electric signal via optical, capacitive, or piezoelectric transducing. Of course, reducing the size of the chambers directly impacts the size of the membrane, and thus its performances, in terms of resonance frequency, detectivity, and signal to noise ratio. Recently, cantilever microphones have been proposed [12,13] and a commercial photoacoustic detection system including a cantilever based solution is available [37].

Quite serious challenges occur in the choice and fabrication of the optical source. One possibility relies on hot filaments. These blackbody sources present a very large bandwidth, at the expense of a low wavelength power density, and must be combined with a high rejection filter in order to address a single gas absorption transition to keep the benefit of the optical sensor selectivity. Moreover, they have a slow operation dynamics. Relying on QCL constitutes an appealing alternative because they are monochromatic and powerful sources, matching closely the needs of the μ-PA sensor. Multi-gas sensing can be addressed, due to the recent development of widely tunable sources constituted by a monolithic array of several tens of distributed feedback QCL [8,10]. Regarding the QCL integration, it is possible to use a separate laser chip in a discrete assembly configuration using for instance lenses [28], an optical fiber [3] or a hollow guide [38] but its coupling with the miniaturized PA cavity or with a waveguide array would negatively impact the LOC properties in terms of compactness, mechanical stability, and cost. Furthermore, using lens alignment to collimate the beam imposes the need to place an antireflective coating at each interface. An attractive alternative consists in directly fabricating the QCL on silicon by heterogeneous integration, but this remains an issue and many technological developments are required in order to transfer and process active laser material on silicon while avoiding cross-contamination between III-V materials and silicon. It can be noted that molecular bonding can be used to transfer QCL epitaxial layers on silicon, as already demonstrated for similar material stacks in silicon photonics for telecommunication applications [39].

Finally, it is important to handle, filter or shape the optical beam from the light source to the acoustic resonant cavity. Compared to free space propagation, integrated photonics is very attractive for this purpose. Silicon photonics, based on silicon on insulator or silicon on sapphire technologies, has proven its efficiency at telecommunication wavelength (1.55 μm) and up to about 5 μm but can not convey light in the whole mid-IR wavelength range of interest (∼3–12 μm) because of silica absorption. Developments targeting a complete CMOS compatible mid-IR photonic platform have been made recently: a waveguide solution involving a variable composition SiGe core in a silicon cladding has shown its capability to produce low loss single mode waveguides up to a wavelength of 8 μm, as well as more complex functions that could prove useful in a fully integrated μ-PA LOC [11].

## 4. Conclusions

The favorable scaling behavior of PA-based gas sensors and the availability of the different components of the system in CMOS-compatible technologies support the idea of developing a LOC μ-PA sensor. For this reason, optical and acoustic models, especially adapted to the LOC size and characteristics, have been devised and coupled to assist in the design of a microdevice currently under fabrication [14].

The question whether further miniaturization would be beneficial is discussed in this paper. The consequences on the physics, models and microfabrication technology are assessed. As chamber downscaling comes with an increase in resonance frequency, the first restriction met when miniaturizing PA sensors is the loss of thermal source modulation due to the competing effects of the periodic excitation of the molecules of interest and their delayed relaxation. The sensibility of the sensor to low concentrations of gases having a long relaxation time would decrease dramatically for microdevices characterized by resonance frequencies above 20–30 kHz. This suggests a limit in miniaturization as the expected signal improvement is, at least partly, cancelled out by the heat source modulation loss.

On the model side, as the working point of the current μ-PA device is close to the limit of validity of the continuum model, and as any size reduction or pressure decrease would bring it even closer, the implementation of velocity slip and temperature jump boundary conditions in the viscothermal model has to be undertaken to help, at least, estimate the influence of the boundary conditions in the μ-PA context. Further improvements of the model can also be expected from the inclusion of the rate equation governing the excitation-relaxation process and the MEMS microphones response and resolution.

On the technological side, even if realizing a complete LOC remains a difficult integration problem, many building blocks such as MEMS microphones and mid-IR waveguides are already available. So far, the main bottleneck is the heterogeneous integration of the mid-IR source in CMOS compatible technologies, which has not yet been reported.

## Figures and Tables

**Table 1.** Relaxation time of several gas molecules on nitrogen at 20 °C and 101,325 Pa.

**Relaxation of**	**CO_2_**	**N_2_O**	**NH_3_**	**NO**
Relaxation time (μs)	11 [23], 7 [26]	1.7 [26]	0.1 [27]	30 [28]

**Table 2.** Characteristic values for the μ-PA cell working point at 20 °C and 101,325 Pa.

$\delta =n^{\overset{-1}{\;}/\underset{3}{\;}}$	**λ** = ***kT***/( $\sqrt{2}\pi nd$)	***L*** = ***d*_μ_**
3.4 nm	66 nm	15 μm
δ/*d*	$Kn\equiv \;\overset{\lambda }{\;}/\underset{L}{\;}$	*L*/δ
9.2	4.4 × 10^−3^	4400
